# Burnout and Biological Biomarkers in Emergency and Acute-Care Healthcare Workers: A Systematic Scoping Review with Evidence Mapping

**DOI:** 10.3390/medicina62030526

**Published:** 2026-03-12

**Authors:** Mihai Alexandru Butoi, Vlad Ionut Belghiru, Monica Iuliana Puticiu, Raluca Tat, Adela Golea, Luciana Teodora Rotaru

**Affiliations:** 1 Emergency Medicine and First Aid Departament, Faculty of Medicine, University of Medicine and Pharmacy, 200349 Craiova, Romania; mihai.butoi@umfcv.ro (M.A.B.); vlad.belghiru@umfcv.ro (V.I.B.); luciana.rotaru@umfcv.ro (L.T.R.); 2 Departamentul de Urgențe, Facultatea de Medicină, Universitatea de Vest Vasile Goldiș din Arad, 310025 Arad, Romania; 3 Surgery Department—Emergency Medicine Discipline, University of Medicine and Pharmacy “Iuliu Hațieganu”, 400347 Cluj, Romania; raluca.tat@umfcluj.ro

**Keywords:** burnout, healthcare workers, emergency department, acute care, biomarkers, cortisol, heart rate variability, inflammation, oxidative stress, stress reactivity, allostatic load, circadian misalignment

## Abstract

*Background and Objectives*: Burnout is highly prevalent among emergency and acute care healthcare workers (HCWs), yet biological correlates remain debated because candidate biomarkers are strongly shaped by circadian timing, shift work, sleep loss, and overlapping affective symptoms. We mapped post-2018 evidence of biological biomarkers assessed alongside validated burnout measures in emergency department (ED), emergency medical services (EMS), and related acute care settings. Specifically, we asked whether reproducible biological correlates of burnout can be identified in emergency and acute-care healthcare workers when biomarker endpoint class and sampling context are systematically considered. *Materials and Methods*: We conducted a systematic scoping review with evidence mapping (PRISMA-ScR). PubMed/MEDLINE and the MDPI platform were searched for English-language studies published from 2018 onward (through January 2026). Eligible quantitative studies enrolled ED/EMS or acute care HCWs, assessed burnout using validated instruments, and reported at least one biological biomarker. Evidence was charted by biomarker domain and endpoint class (basal measures, stress reactivity paradigms, and chronic indices such as hair-based markers). *Results*: Overall, 19 studies were included in mapping/synthesis. Biomarker selection clustered around the hypothalamic–pituitary–adrenal axis (cortisol; n = 10/19), with fewer studies focused on autonomic function (heart rate variability; n = 2/19) and immune–inflammatory markers (n = 2/19), and single-study coverage for oxidative stress (n = 1/19), cardiometabolic candidates (n = 1/19), cellular aging (n = 1/19), neuroglial/multi-system candidates (n = 1/19), and feasibility-oriented multi-marker designs (n = 1/19). Reported associations with burnout were heterogeneous in direction and magnitude, but were more interpretable when endpoint class, timing anchors, and shift/sleep-related covariates were explicitly reported. Rates of confounder adjustment were low across studies (e.g., only 3/19 reported multivariable adjustment, and none systematically measured sleep or circadian factors), substantially limiting interpretability. *Conclusions*: The 2018+ literature does not support a single reproducible biomarker for burnout in emergency and acute care workforces. Evidence instead suggests multi-system dysregulation that is highly sensitive to endpoint class, sampling timing, and contextual confounding. Future studies should prioritize timing-anchored repeated-measures protocols across shift and recovery windows, jointly model sleep/circadian factors and depressive symptoms, and evaluate multi-marker panels and intervention responsiveness.

## 1. Introduction

Emergency and acute care clinicians operate at the intersection of time-critical decisions, high cognitive load, and repeated exposure to trauma, death, and ethical complexity. These demands coincide with rotating shifts, sleep debt, and circadian misalignment, creating a biological milieu in which stress-system activation may become persistent rather than episodic. Across settings, emergency physicians and ED nurses are consistently reported to carry a high burnout burden, with downstream implications for retention, team functioning, and patient safety [1,2]. In practice, burnout is rarely a purely individual phenomenon: it is shaped by workload intensity, staffing and skill mix, clinical uncertainty, and the moral weight of constrained resources in the face of urgent need.

In Romania, emergency medicine has undergone rapid maturation over the past three decades, accompanied by growing empirical attention to workforce wellbeing and organizational strain. Recent studies in Romanian emergency physicians, residents, and prehospital emergency personnel consistently report substantial burnout burden and highlight the interplay with work addiction and stress-related growth, suggesting setting-specific psychosocial and system drivers relevant to emergency and acute care workforces. These findings strengthen the rationale for integrating biomarker-informed phenotyping with validated burnout measures when studying high-intensity, shift-working clinical environments [3,4,5,6].

Clinically, the relevance is twofold. First, burnout can degrade attentional control, situational awareness, and communication in environments where errors have rapid consequences, potentially amplifying risk in resuscitation, triage, and high-acuity transitions. Second, burnout is associated with health and functioning of the workforce itself, including sleep disturbance and stress-related somatic complaints, which may in turn reinforce adverse scheduling cycles and absenteeism. Acute care ecosystems are therefore exposed to feedback loops: as staffing becomes more fragile, workload rises, recovery opportunities narrow, and the probability of sustained strain increases.

In ICD-11, burnout is framed as an occupational phenomenon rather than a medical diagnosis, characterized by exhaustion, increased mental distance or cynicism toward one’s job, and reduced professional efficacy [7]. This distinction matters clinically because overlapping symptom language may reflect depression, anxiety, post-traumatic stress, or sleep-related disorders, and the field continues to debate whether burnout constitutes a separable syndrome or largely overlaps with broader distress [8,9]. Measurement choices further complicate interpretation: studies differ in instrument selection, dimensional versus categorical operationalization, and cut-offs, making it difficult to determine whether biological correlates map to burnout specifically, to general distress, or to co-occurring conditions.

Biomarker research has been proposed as a partial remedy to this ambiguity by providing objective correlates of stress-system dynamics that complement self-report. Candidate pathways align with clinical stress medicine: HPA-axis and circadian markers (notably hair or salivary cortisol), autonomic regulation (heart rate variability), inflammatory signaling (cytokines and related immune markers), oxidative stress indices, cardiometabolic risk markers, and cellular aging measures (telomere-related outcomes). Mechanistic frameworks such as allostatic load theory provide a coherent rationale, conceptualizing cumulative ‘wear and tear’ across neuroendocrine, autonomic, immune, and metabolic systems when adaptive responses are repeatedly activated or insufficiently terminated [10,11]. From this perspective, a single universal biomarker signature is unlikely; instead, biomarkers may serve as pathway-level indicators that vary by exposure intensity, recovery opportunities, and individual vulnerability. This theoretical framework directly informed our evidence-mapping strategy, which organizes findings by physiological domain and endpoint class (basal regulation, stress reactivity paradigms, and chronic load indices), thereby reflecting distinct layers of allostatic adaptation and dysregulation.

However, emergency and acute care contexts amplify interpretive challenges. Many biomarkers exhibit strong diurnal rhythms and sensitivity to shift timing, sleep loss, medication use, intercurrent infection or inflammation, and adiposity. As a result, studies that do not specify sampling windows, shift phase, or recovery time risk conflating circadian disruption with burnout biology. Endpoint non-equivalence is common: basal levels, chronic indices (e.g., hair cortisol), and stress-reactivity readouts capture different processes and should not be interpreted interchangeably.

From a clinical standpoint, the promise of biomarker work is twofold. First, it may help distinguish transient fatigue from sustained dysregulation, identifying staff at heightened risk for downstream cardiometabolic or affective complications. Second, biomarkers can provide objective endpoints for evaluating organizational or individual-level interventions when self-report is limited by stigma or differential response patterns.

Given these constraints and the rapid expansion of biomarker modalities (including wearable autonomic monitoring), an evidence map is needed to clarify what has been measured, in which populations, and with which protocols. We therefore performed a systematic scoping review with evidence mapping of English-language studies published since 2018 that assessed burnout using validated instruments and reported biological biomarkers in emergency and related acute care healthcare workers. We summarized biomarker domains, sampling paradigm, and confounding adjustment, and we highlight gaps that must be addressed before quantitative synthesis or clinical translation is feasible [12].

To date, no review has systematically mapped biological biomarkers of burnout specifically within emergency and acute-care healthcare workers while explicitly distinguishing endpoint classes (basal measures, stress reactivity paradigms, and chronic load indices). Existing reviews have discussed biomarkers in broader occupational samples or without structured differentiation of sampling timing and physiological endpoint class. This gap limits interpretive clarity, particularly in shift-disrupted emergency settings. The present review therefore aims to provide a structured domain-by-endpoint evidence map to clarify heterogeneity and methodological constraints.

## 2. Materials and Methods

### 2.1. Design and Reporting

**Figure 1:**
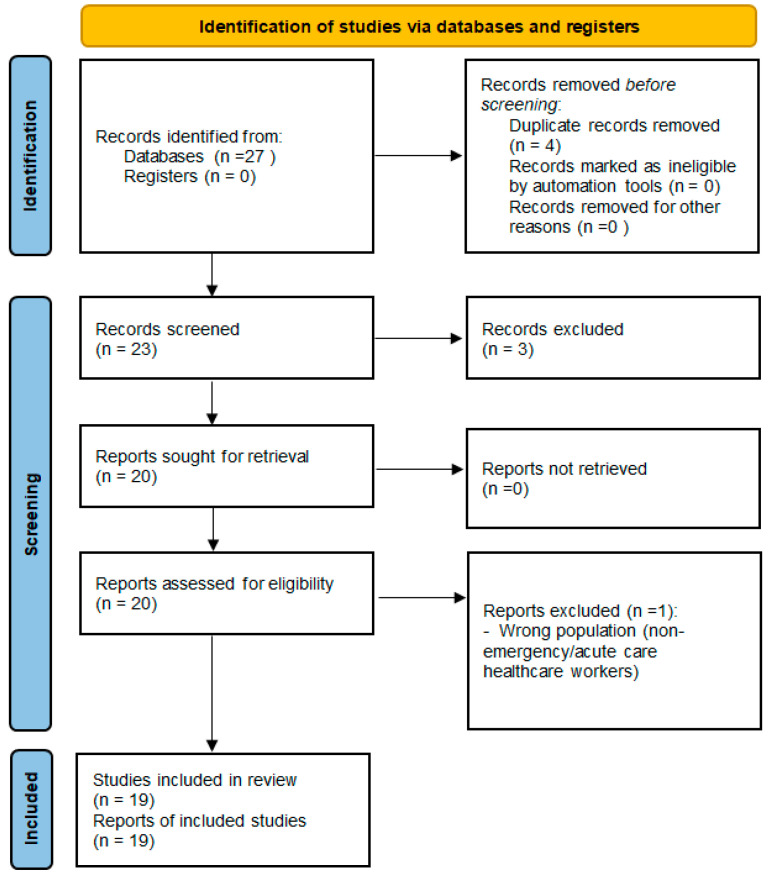
PRISMA 2020 flow diagram of study selection. Context-only sources were retained outside PRISMA counts and used for methodological contextualization only.

We conducted a systematic scoping review with evidence mapping and critical narrative synthesis focused on links between validated burnout measures and biological biomarkers in emergency and related acute care healthcare workers (HCWs). Reporting followed Preferred Reporting Items for Systematic Reviews and Meta-Analyses (PRISMA)-ScR [12], and study selection is summarized in a PRISMA 2020-style flow diagram (Figure 1) [13].

### 2.2. Eligibility Criteria

We included original quantitative studies (observational or interventional) enrolling HCWs working in emergency and/or related acute care settings (ED/emergency medical services (EMS)/intensive care unit (ICU) or mixed acute care hospital settings) that assessed burnout using a validated instrument and reported at least one biological biomarker (blood, saliva, hair, urine, or wearable-derived physiology such as heart rate variability). We excluded non-English records, studies published before 2018, non-original publications, non-HCW populations, and studies without a validated burnout construct.

### 2.3. Information Sources and Search Strategy

We performed searches in PubMed/MEDLINE and the MDPI platform from 1 January 2018 to the final search date. The strategy combined terms for burnout (including instrument terms), HCWs, emergency and acute care contexts, and biomarker domains (HPA-axis/cortisol, autonomic function/heart rate variability, inflammatory markers, oxidative stress, cardiometabolic markers, and cellular aging). Reference lists of included studies were also screened. In PubMed, controlled vocabulary (MeSH terms) was systematically combined with free-text keywords (title/abstract fields) using Boolean operators to enhance sensitivity and specificity. Search blocks covered burnout constructs, emergency/acute-care populations, healthcare worker descriptors, and biomarker-related terms. Full reproducible search strategies (complete query strings for each information source) are provided in the Supplementary Materials (Table S2). The restriction to studies published from 2018 onward was applied to capture contemporary biomarker methodologies and validated burnout instruments, reflecting recent advances in psychoneuroendocrine and immunological assessment as well as improved standardization of reporting. Earlier literature often employed heterogeneous measurement paradigms and less comparable sampling and assay workflows. The end date corresponds to the last search update within the study timeline.

### 2.4. Study Selection

Records were deduplicated and screened at title/abstract level, followed by full-text assessment against prespecified criteria; reasons for full-text exclusion were recorded. Title/abstract screening and full-text assessment were performed independently by two reviewers. Discrepancies were resolved through discussion, with consensus reached in all cases. For the final 2018+ evidence map, records identified (n = 27), duplicates removed (n = 4), records screened (n = 23), excluded at title/abstract (n = 3), full-text assessed (n = 20), full-text excluded (n = 1), and studies included in mapping/synthesis (n = 19). Context-only sources (n = 5) were retained outside PRISMA.

The overall workflow of screening, data charting, and evidence mapping is summarized in Figure 1.

### 2.5. Data Charting and Synthesis

We charted study-level data using a prespecified template capturing setting and participants, burnout instrument and operationalization, biomarker domain(s), specimen and timing (basal, chronic load, and/or stress reactivity), statistical approach, and confounding control. Evidence mapping summarized biomarker coverage by domain and sampling paradigm (Table 1) and tracked construct, protocol, and confounding patterns (Table 2). Findings were synthesized narratively with emphasis on endpoint timescale and shift/circadian validity.

### 2.6. Risk-of-Bias Appraisal

Risk of bias was appraised to contextualize confidence in findings rather than to exclude studies. Joanna Briggs Institute (JBI) checklists were used for analytical cross-sectional designs [14], the Newcastle-Ottawa Scale for cohort studies [17], and Risk Of Bias In Non-randomized Studies of Interventions (ROBINS-I) for non-randomized interventions [15]. Judgements were reported by domain and used to temper inference. Specifically, narrative synthesis emphasized study design robustness, degree of confounder adjustment (particularly for sleep, shift timing, depressive symptoms, and medication use), and clarity of sampling protocols. Findings from studies with limited adjustment or poorly specified timing anchors were interpreted with greater caution.

## 3. Results

Study selection is summarized in Figure 1. Using the predefined criteria (English; published ≥ 2018), we identified 27 records, removed 4 duplicates, and included 19 studies in the evidence map (Table 1 and Table 2). Context-only sources (n = 5) were used to contextualize biomarker protocols and confounding patterns but were not entered into PRISMA counts.

**Figure 2:**
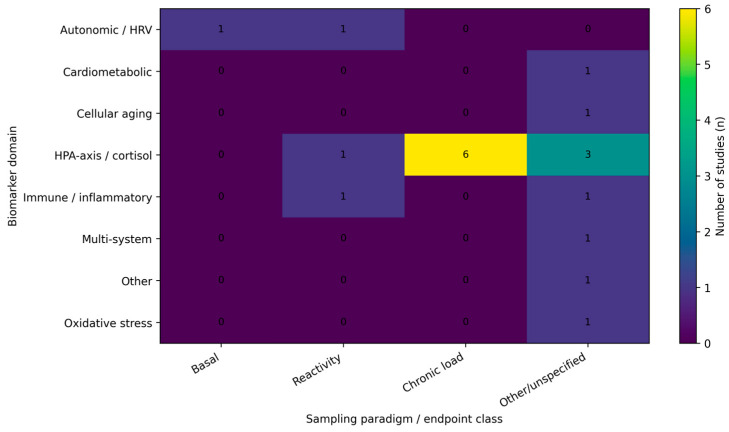
Evidence map heatmap of biomarker domains by sampling paradigm. Cells show the number of included studies (n) within each biomarker domain that reported basal measures, stress reactivity outcomes, chronic indices (e.g., hair cortisol), or other/unspecified endpoint classes. When studies included multiple biomarkers, classification was based on the dominant analytic domain as defined in the evidence map.

**Figure 3:**
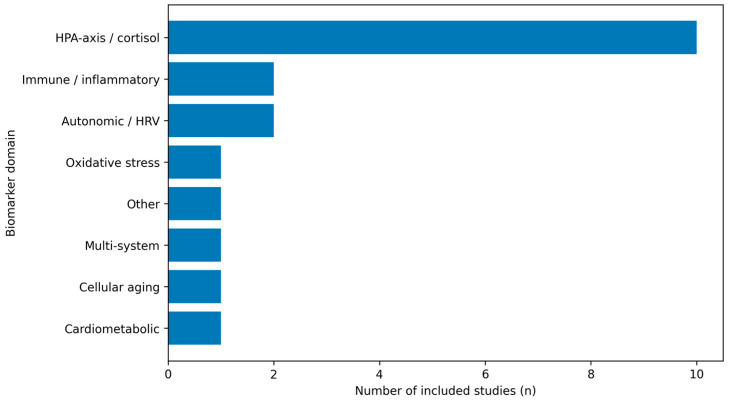
Distribution of included studies by primary biomarker domain. For mapping purposes, each included study was assigned to a single dominant biomarker domain, even when multiple biomarkers were measured; assignment reflected the central analytic focus of the study (total n = 19).

Across included studies, biomarker selection broadly reflects stress-system frameworks, with emphasis on HPA-axis/cortisol dynamics, autonomic regulation (heart rate variability), and immune–inflammatory markers, and less frequent use of oxidative stress, cardiometabolic, cellular aging, or neuroglial candidates. The empirical landscape remains fragmented, however, because studies differ not only in biomarker domain coverage but also—critically—in the timescale indexed by the biomarker endpoint (basal levels, chronic indices, or stress reactivity). We therefore present an evidence map summarizing (i) endpoint classes by biomarker domain (Figure 2), (ii) the distribution of studies across primary biomarker domains (Figure 3), and (iii) study-level details by domain, protocol, and setting (Table 1).

Endpoint non-equivalence was a major source of heterogeneity: basal samples, provoked stress-reactivity protocols, and chronic indices (e.g., hair cortisol) capture different biological processes and should not be interpreted interchangeably. Figure 2 summarizes how these endpoint classes were distributed across biomarker domains in the included studies.

Figure 3 provides a complementary view, showing the distribution of included studies across the primary biomarker domains used in the evidence map.

Three observations recur. First, results are highly sensitive to sampling design: diurnal timing, shift context, and whether outcomes index acute reactivity versus longer-term embedding. Second, burnout phenotyping varies widely (instrument choice, dimensionality, and cut-offs), limiting comparability and increasing the risk that different studies are not estimating the same construct [16]. Third, associations are difficult to interpret without concurrent measurement of depression, anxiety, fatigue, and sleep—domains that overlap strongly with burnout and can plausibly mediate or confound biomarker relationships in emergency settings [8,9]. This limitation is also reflected quantitatively in Table 2: only 1/19 studies (5.3%) explicitly reported shift work/rotating schedules, none reported measuring or controlling sleep (0/19, 0.0%), and only 3/19 studies (15.8%) reported multivariable adjustment. These very low rates of confounder control substantially constrain interpretability across biomarker domains.

**Figure 4:**
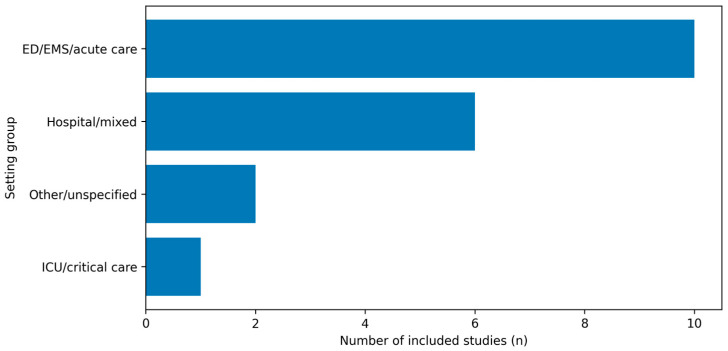
Distribution of included studies by clinical setting. Studies were grouped as ED/EMS/acute care, mixed hospital acute-care settings, ICU/critical care, or other/unspecified, based on the setting classification used in the evidence map.

Most included studies were conducted in ED/EMS-focused cohorts, with fewer studies in mixed hospital acute-care settings and ICU-only samples. The distribution of included studies by clinical setting is shown in Figure 4.

### 3.1. Hypothalamic–Pituitary–Adrenal (HPA) Axis Measures

Cortisol is the most frequently studied biomarker in burnout research, but also among the most method-sensitive. Studies have used single morning samples, diurnal slopes, the cortisol awakening response (CAR), hair cortisol concentration (HCC) as an integrated index, and cortisol reactivity to standardized psychosocial stress. Experimental work shows that acute HPA responses are shaped by sex hormones, age, prior stress exposure, and the stress task protocol itself [19].

In emergency care personnel, shift work and sleep restriction create two interpretive traps. Apparent “hypocortisolism” may reflect circadian phase shifts rather than downregulated HPA function; and single samples may capture transient post-shift states rather than chronic dysregulation. The broader burnout literature has oscillated between models of HPA hyperactivation in earlier phases and blunting in prolonged or severe burnout, with no consensus [18]. Hair cortisol is practically attractive for emergency staff because it integrates weeks-to-months exposure and is less sensitive to immediate shift timing, but occupational scoping reviews highlight substantial standardization challenges (hair treatment, sampling site, growth-rate assumptions) and limited specificity of HCC for psychosocial stress [20,21].

Recent physician research using standardized psychosocial stress testing indicates that altered cortisol reactivity can be detectable even when basal indices are equivocal, which supports a focus on dynamic measures (reactivity and recovery) rather than static levels alone [22]. Whether such dynamic HPA phenotypes reliably distinguish burnout from depression, sleep loss, or other stress-related conditions remains an unresolved question.

### 3.2. Autonomic Regulation and Heart Rate Variability (HRV)

HRV is commonly used as a non-invasive index of autonomic regulation, with lower vagally mediated HRV often interpreted as reduced parasympathetic (recovery) capacity. Conceptual work links vagal function to emotion regulation and stress resilience [23]. In emergency settings, HRV is attractive because wearables can capture rapid physiological shifts during real clinical work, enabling within-person stress-recovery modeling.

However, HRV is sensitive to posture, respiration, caffeine and nicotine, physical activity, and measurement artifacts, all common in ED workflows. A recent systematic review and meta-analysis in doctors supports the feasibility of continuous ambulatory HRV monitoring to capture stress and recovery patterns relevant to burnout, but emphasizes heterogeneity in devices, signal processing, and analytic choices, and limited standardization of burnout phenotyping [24]. Consequently, current HRV findings are best interpreted as markers of autonomic strain and impaired recovery that may accompany burnout, rather than diagnostic indicators.

### 3.3. Inflammatory and Immune Biomarkers

The hypothesis that burnout contributes to low-grade inflammation fits within allostatic load models, but empirical evidence is mixed. In large samples outside emergency medicine, burnout, depression, and anxiety are differentially associated with microinflammation markers such as hs-CRP and fibrinogen, with sex-specific patterns [25]. Occupational health evidence also supports associations between adverse working conditions and inflammatory biomarkers, including prospective data, although effect sizes are modest and residual confounding remains a major concern [26].

In physician samples, experimental paradigms increasingly assess immune reactivity rather than basal levels. Altered interleukin-6 (IL-6) stress reactivity has been reported in male physicians with occupational burnout [27], supporting a dysregulated response interpretation consistent with increased allostatic load. Such findings are mechanistically informative but intensify a key specificity debate: are immune differences attributable to burnout, or to correlated exposures such as sleep loss, infections, metabolic risk factors, or depressive symptom burden? Emergency-specific studies rarely measure these confounders comprehensively, limiting causal claims.

### 3.4. Oxidative Stress and Metabolic Risk Markers

Oxidative stress biomarkers (e.g., malondialdehyde and antioxidant enzymes) and metabolic indices (lipid and glucose-related markers) appear intermittently in the emergency burnout literature, usually motivated by concerns that chronic occupational stress may accelerate cardiometabolic risk. Yet these measures are among the least specific: diet, physical activity, smoking, acute infection, and circadian disruption have large effects. For shift-working emergency personnel, biomarkers may therefore capture physiological costs of circadian disruption as much as, or more than, burnout itself.

Most emergency-focused studies in this domain are small and cross-sectional, vulnerable to multiple-comparison findings and residual confounding. Nevertheless, incorporating oxidative and metabolic markers remains conceptually coherent if burnout is treated as co-occurring with multi-system physiological strain rather than as a single-pathway disorder [11,28].

### 3.5. Neuroglial Candidates: S100B

S100B has been examined as a peripheral candidate biomarker linked to glial activation and, in other contexts, to brain injury and affective symptoms. In emergency medicine residents, serum S100B has been reported to correlate with depression scores and emotional exhaustion [29]. Subsequent ED staff studies have explored similar associations, although samples are often limited and covariate control varies [30].

Two interpretive caveats are central. First, S100B is not specific to psychosocial stress and may be influenced by physical exertion and subclinical neurological insults. Second, the biological meaning of modest peripheral differences is without convergent evidence from neurocognitive outcomes or multi-marker panels. At present, the most defensible conclusion is that S100B remains hypothesis-generating rather than validated as a biomarker of burnout.

Unlike cortisol or inflammatory cytokines, S100B is conceptually linked to glial activation and blood–brain barrier permeability, placing it closer to neurobiological stress integration rather than peripheral stress physiology alone. This theoretical proximity to central nervous system processes makes S100B biologically intriguing in burnout research, but also heightens the need for convergent validation through neurocognitive measures or multi-marker panels.

To maintain structural clarity, the Results section reports descriptive domain patterns and study-level associations; broader interpretative synthesis and translational implications are developed in the Discussion.

## 4. Discussion

### 4.1. Areas of Convergence

Across the 2018+ literature, burnout research in emergency and acute-care healthcare workers converges on a multi-system interpretation rather than a single “signature” marker. Included studies most frequently targeted the hypothalamic–pituitary–adrenal axis (cortisol in saliva/serum or hair) and autonomic regulation (heart rate variability), with smaller clusters in immune–inflammatory markers and less frequent use of oxidative stress, cardiometabolic, or cellular aging candidates (Table 1; Figure 2 and Figure 3). Taken together, this pattern is compatible with stress-system and allostatic load frameworks, in which prolonged occupational strain manifests across coordinated physiological axes rather than as an isolated biomarker change [10,11].

A second area of convergence concerns interpretability: authors repeatedly emphasize that biomarker signals are highly sensitive to timing, sleep and shift patterns, and acute intercurrent conditions. This is especially salient in emergency department and emergency medical services contexts where exposure intensity fluctuates within and across shifts and recovery windows may be truncated. In practical terms, a biomarker value is only comparable across individuals and studies when sampling windows are anchored to explicit timing information (clock time and/or wake time), and when key acute modifiers (recent sleep, stimulants, infection, and physical exertion) are measured and transparently reported.

Finally, the evidence base converges on the need for protocol transparency and harmonization. Even when findings are internally consistent within a given design, incomplete reporting of sampling windows, assay characteristics, and covariate handling limits cross-study comparability. The construct–protocol–confounding matrix (Table 2) suggests that a shared minimum reporting set would materially strengthen inference and reduce apparent contradictions that may be methodological rather than biological.

### 4.2. Burnout Versus Depression and Shared Distress

Construct overlap remains a key source of disagreement: burnout shares symptom space with depression, anxiety, and generalized distress, and instruments variably operationalize the syndrome (multi-dimensional scales, abbreviated versions, or single-item screens). This matters biologically because many candidate biomarkers are not disorder-specific; they may index transdiagnostic stress, sleep debt, or inflammatory activation rather than burnout per se. Accordingly, a recurring debate is whether biomarker associations reflect burnout specifically or broader distress processes that co-occur with burnout symptoms [8,9].

A pragmatic implication is that “burnout–biomarker” associations should be interpreted as conditional on construct specification. Associations may differ depending on whether analyses focus on exhaustion versus cynicism or professional efficacy, whether symptoms are modeled dimensionally or by cut-offs, and whether depressive symptoms are measured and adjusted. When depression is not assessed or not incorporated analytically, residual confounding can inflate perceived specificity; conversely, overly aggressive adjustment can remove the shared variance that is mechanistically relevant if burnout is conceptualized within a broader distress spectrum.

Our oriented translational framing, the key question is less whether burnout is perfectly separable from depression at the biomarker level and more whether biologically informed phenotyping can improve risk stratification and guide prevention in emergency and acute-care workforces. Future studies should therefore routinely collect parallel measures of depressive symptoms and sleep impairment and report sensitivity analyses that clarify whether biological signals track burnout dimensions after accounting for these overlapping constructs.

Beyond construct overlap with depression, a deeper epistemological asymmetry warrants consideration. Burnout is fundamentally shaped by organizational, relational, and moral dimensions of work that may not necessarily manifest as stable or detectable biological signatures. Conversely, biological dysregulation related to sleep deprivation, circadian disruption, or metabolic stress does not inevitably translate into subjective burnout if protective psychosocial factors are present. This asymmetry underscores a core limitation of biomarker-driven approaches: physiological correlates may reflect stress-related processes without fully capturing the experiential and existential dimensions of burnout. Accordingly, biomarkers should be interpreted as context-sensitive physiological indicators rather than diagnostic substitutes for a multidimensional occupational construct.

### 4.3. Reactivity Versus Basal Levels and the Timescale Problem

Endpoints are often treated as equivalent when they are not. Basal samples, provoked stress-reactivity protocols, and chronic indices (such as hair cortisol) capture different timescales and biological processes and should not be interpreted interchangeably. Figure 2 makes this explicit by showing that endpoint classes are mixed across biomarker domains in the included studies, which can yield apparently inconsistent findings even when underlying physiology is coherent.

In emergency and acute-care contexts, circadian misalignment is central. Cortisol has strong diurnal structure, and autonomic measures are sensitive to posture, recent physical activity, caffeine or nicotine exposure, and sleep loss. A single basal sample obtained after a night shift is not comparable to one drawn after regular sleep, and a single resting heart rate variability snapshot may reflect acute fatigue rather than chronic strain. Thus, null findings or directionally mixed results may reflect timing noise and acute-state contamination rather than the absence of a true association with burnout dimensions.

Methodologically, designs that incorporate repeated measures across shift phases and recovery days, paired with explicit timing anchors (clock time, wake time, and shift timing), are better aligned to the biology of reactivity and recovery. Chronic indices may be advantageous when the aim is cumulative load, whereas task-based or shift-based paradigms may better capture dysregulated responsiveness. Interpreting biomarker findings therefore requires matching the endpoint to the hypothesized mechanism and adopting analysis strategies that separate within-person dynamics (shift-to-shift variation) from between-person differences (trait-like vulnerability or resilience).

### 4.4. Specificity, Confounding, and Clinical Utility

Specificity is the central translational challenge because most candidate biomarkers are influenced by strong non-burnout determinants. Shift schedules, sleep duration and quality, body composition and metabolic status, physical fitness, smoking, medication use, intercurrent infection or inflammation, and menstrual status can all materially shift cortisol, heart rate variability, and inflammatory markers. In acute-care cohorts, organizational variables (staffing, workload intensity, break opportunities, and moral or organizational stressors) may act as both exposures and modifiers, complicating causal interpretation when they are unmeasured or only crudely approximated.

Table 2 indicates that many studies adjust for only a subset of these factors, often due to sample size constraints or incomplete exposure measurement. This pattern suggests that the most useful near-term role of biomarkers is unlikely to be “diagnosing” burnout. Rather, biomarkers can support (i) mechanistic interpretation, (ii) identification of higher-risk phenotypes (for example, impaired recovery signatures across shifts), and (iii) objective monitoring of response to organizational or individual-level interventions—particularly when combined with brief validated burnout screens and clear exposure metrics. This positioning is consistent with a monitoring and evaluation model, and aligns with the interventional studies included in the evidence base [22,31,32].

For clinical and occupational health practice, an incremental utility model is therefore appropriate: biological measures should be treated as risk modifiers within a multi-source assessment that includes validated questionnaires, sleep and shift metrics, and—where feasible—organizational exposure indicators. Ethical implementation also requires explicit governance emphasizing worker benefit, confidentiality, and avoidance of punitive use. In this framing, biomarkers can inform prevention strategies and help evaluate whether interventions improve recovery biology alongside self-reported burnout.

Review-Level Limitations

An additional methodological limitation concerns the restriction of database searching to PubMed/MEDLINE and the MDPI platform. Although PubMed indexes a substantial proportion of biomedical literature, the absence of Embase, PsycINFO, and Web of Science/Scopus may have limited coverage of interdisciplinary and psychology-focused studies. The inclusion of the MDPI platform reflects the journal ecosystem in which several eligible studies were published, but we acknowledge that it partially overlaps with PubMed indexing and does not substitute for broader database coverage. This restriction may introduce selection bias and limits the comprehensiveness typically expected of larger scoping reviews. Findings should therefore be interpreted within this constraint.

The relatively small number of references reflects the genuinely limited number of eligible empirical studies meeting the predefined inclusion criteria (i.e., assessment of biological biomarkers alongside validated burnout measures in emergency and acute-care healthcare workers). Of the screened literature, only 19 studies fulfilled these criteria. Thus, the scope of cited evidence represents the size of the available domain rather than selective reporting.

### 4.5. Evidence Gaps and Priority Research Questions

Key gaps include limited emergency department and emergency medical services-focused longitudinal evidence, scarce studies that jointly model burnout, sleep and circadian misalignment, depressive symptoms, and limited harmonization of sampling protocols and confounder sets. Although the evidence base has expanded since 2018, a substantial fraction of studies still derive findings from mixed acute-care settings, which may not fully reflect the distinctive exposure profile of emergency care clinicians (Figure 4).

Priority research questions therefore include: (1) which biomarker endpoints best capture impaired recovery and cumulative load in shift-working acute-care clinicians; (2) whether burnout dimensions (exhaustion versus cynicism or professional efficacy) map onto distinct biological patterns when depression and sleep impairment are measured concurrently; and (3) whether biomarker-informed phenotyping adds predictive value for outcomes that matter to health systems and patient care (such as sickness absence, retention, self-reported errors, and patient-safety proxies). Importantly, these questions require study designs that can separate acute-state effects from cumulative effects and can account for organizational exposures as upstream determinants.

Methodologically, future work would benefit from a minimum core protocol: standardized timing anchors, repeated sampling across shift phases and recovery days, transparent assay and preprocessing reporting, and a prespecified confounder set (at minimum: age, sex, body mass index, smoking, key medications, sleep metrics, and shift characteristics). Interventional studies (including randomized controlled trials or quasi-experiments) that include both burnout outcomes and biomarker endpoints are especially valuable because they can test whether changes in organizational or behavioral exposures translate into measurable biological recovery, thereby strengthening causal interpretation.

Earlier biomarker-focused syntheses in occupational burnout have emphasized that apparent contradictions across studies often reflect heterogeneity in constructs, sampling paradigms, and covariate handling rather than stable biological null effects. Systematic and narrative reviews have highlighted the sensitivity of endocrine and immune readouts to timing and contextual factors and, therefore, the importance of explicit protocol reporting and confounder control when interpreting cortisol-, inflammatory-, and related outcomes in burnout research [31,32].

Recent chronobiology-oriented work further strengthens this interpretation by framing burnout-related hormonal dysregulation through the lens of circadian misalignment and biological timing. In shift-working cohorts, complementary assessment of HPA-axis markers (e.g., cortisol) alongside circadian phase–linked measures (e.g., melatonin) may improve interpretability, and key zeitgebers such as light exposure should be treated as protocol determinants rather than background noise [33]. Taken together, these perspectives support our evidence-mapping approach: when constructs and endpoints are non-equivalent across domains, structured domain-by-endpoint mapping and transparent, heterogeneity-aware synthesis may be more informative than premature quantitative pooling.

In synthesis, priority directions emerging from this evidence map include the adoption of standardized timing anchors and repeated-measures designs across shift and recovery windows; concurrent modeling of burnout dimensions alongside depressive symptoms and sleep or circadian variables; implementation of prespecified confounder sets with transparent assay reporting; evaluation of multi-marker panels rather than reliance on single biomarkers; and integration of biological endpoints into interventional and quasi-experimental designs to strengthen causal inference.

## 5. Conclusions

Burnout in emergency and acute care healthcare workers is a high-burden occupational phenomenon with plausible biological correlates across neuroendocrine (HPA-axis), autonomic, and immune–inflammatory systems. However, this scoping review with evidence mapping indicates that findings differ substantially by biomarker domain and endpoint class (basal measures, stress reactivity paradigms, and chronic indices), and no single biomarker is sufficiently reproducible or specific to support diagnostic use. The most coherent interpretation is consistent with allostatic load in shift-working environments, in which circadian misalignment and sleep loss shape multisystem dysregulation and reduce physiological flexibility. Progress will require timing-anchored and transparently reported protocols, longitudinal within-person designs, prespecified confounder sets (including sleep/circadian measures and overlapping affective symptoms), and evaluation of multi-marker panels and intervention responsiveness. By structuring the literature through a domain-by-endpoint evidence-mapping framework, this review clarifies sources of apparent heterogeneity and provides a methodological foundation for more coherent future synthesis and translational research in emergency and acute-care settings.

## Figures and Tables

**Table 1:** Evidence map by biomarker domain, sampling paradigm, and setting across included studies (n = 19).

Biomarker Domain	Typical Biomarkers/Specimens (Examples)	Sampling Paradigm(s) Reported	Settings Represented	Studies, n (Ref. Nos)
Autonomic/HRV	ECG-derived HRV features; HRV	Basal (n = 1); Reactivity (n = 1)	ED/EMS (n = 2)	2 (Refs.: [3,10])
Cardiometabolic	Waist circumference	Other/unspecified (n = 1)	ED/EMS (n = 1)	1 (Refs.: [1])
Cellular aging	Leukocyte telomere length	Other/unspecified (n = 1)	ED/EMS (n = 1)	1 (Refs.: [12])
HPA-axis/cortisol	Hair cortisol; Hair cortisol (stress reactivity index); Hair cortisol change; Hair cortisol concentration (HCC); Sali.	Reactivity (n = 1); Chronic load (n = 6); Other/unspecified (n = 3)	ED/EMS (n = 10)	10 (Refs.: [2,4,5,6,7,9,11,14,15,16])
Immune/inflammatory	IL-6 stress reactivity (acute psychosocial stress test); Protein and lipid oxidative damage (e.g., TBARS)	Reactivity (n = 1); Other/unspecified (n = 1)	ED/EMS (n = 1); ICU (n = 1)	2 (Refs.: [17,18])
Multi-system	HRV	Other/unspecified (n = 1)	ED/EMS (n = 1)	1 (Refs.: [19])
Other	Multiple biomarkers (feasibility)	Other/unspecified (n = 1)	ED/EMS (n = 1)	1 (Refs.: [13])
Oxidative stress	MDA	Other/unspecified (n = 1)	ED/EMS (n = 1)	1 (Refs.: [8])

**Table 2:** Summary of construct operationalization, biospecimen protocol, and confounding control across included studies (n = 19).

Reporting Domain	Category	n/N (%)	Notes
Study design	Observational	14/19 (73.7%)	
Study design	Interventional	5/19 (26.3%)	
Burnout operationalization	MBI family	12/19 (63.2%)	
Burnout operationalization	Other validated multi-item	1/19 (5.3%)	
Burnout operationalization	Single-item (Mini-Z/other)	1/19 (5.3%)	
Burnout operationalization	NR	5/19 (26.3%)	NR, not reported.
Biomarker sampling paradigm	Basal	1/19 (5.3%)	
Biomarker sampling paradigm	Chronic load	6/19 (31.6%)	
Biomarker sampling paradigm	Reactivity	3/19 (15.8%)	
Biomarker sampling paradigm	Pre/post	1/19 (5.3%)	
Biomarker sampling paradigm	Other specified	8/19 (42.1%)	
Biomarker sampling paradigm	NR	0/19 (0.0%)	NR, not reported.
Key covariates/confounding control	Shift work/rotating schedule	1/19 (5.3%)	Based on explicit reporting in included papers; NR treated as not reported.
Key covariates/confounding control	Sleep measured/controlled	0/19 (0.0%)	Based on explicit reporting in included papers; NR treated as not reported.
Key covariates/confounding control	BMI/adiposity	0/19 (0.0%)	Based on explicit reporting in included papers; NR treated as not reported.
Key covariates/confounding control	Smoking/caffeine/nicotine	0/19 (0.0%)	Based on explicit reporting in included papers; NR treated as not reported.
Key covariates/confounding control	Medication/illness noted	0/19 (0.0%)	Based on explicit reporting in included papers; NR treated as not reported.
Key covariates/confounding control	Multivariable adjustment reported	3/19 (15.8%)	Based on explicit reporting in included papers; NR treated as not reported.

## Data Availability

No new data were created or analyzed in this study. Data sharing is not applicable.

## Supplementary Materials

The following supporting information can be downloaded at: https://www.mdpi.com/article/10.3390/medicina62030526/s1, Table S1: PRISMA 2020 checklist; Table S2: Full reproducible search strategies.

## Abbreviations

The following abbreviations are used in this manuscript:

CAR	Cortisol Awakening Response
ED	Emergency Department
EMS	Emergency Medical Services
HCC	Hair Cortisol Concentration
HCWs	Healthcare Workers
HPA	Hypothalamic–Pituitary–Adrenal
HRV	Heart Rate Variability
hs-CRP	High-Sensitivity C-Reactive Protein
ICD-11	International Classification of Diseases, 11th Revision
ICU	Intensive Care Unit
IL-6	Interleukin-6
JBI	Joanna Briggs Institute
MEDLINE	Medical Literature Analysis and Retrieval System Online
PRISMA	Preferred Reporting Items for Systematic Reviews and Meta-Analyses
PRISMA-ScR	PRISMA Extension for Scoping Reviews
ROBINS-I	Risk Of Bias In Non-randomized Studies of Interventions
