# Supplementary material for: Burnout and Biological Biomarkers in Emergency and Acute-Care Healthcare Workers: A Systematic Scoping Review with Evidence Mapping
Source: Medicina (Kaunas). 2026 Mar 12;62(3):526. doi: 10.3390/medicina62030526 (PMC13027818; doi:10.3390/medicina62030526)
Supplement: Supplementary file 1 [file medicina-62-00526-s001.zip › medicina-4182520-supplementary.pdf]

Supplementary Materials

Table S1. PRISMA 2020 Checklist with Manuscript Locations.

**Section / Topic**	**Item**	**PRISMA 2020 Checklist Item**	**Location in Manuscript**
Title	1	Identify the report as a systematic review.	Title page
Abstract	2	Provide a structured summary including background, objectives, methods, results, and conclusions.	Abstract
Introduction	3	Describe the rationale for the review in the context of existing knowledge.	Section 1
Introduction	4	Provide an explicit statement of the objective(s) or question(s) addressed.	Section 1
Methods	5	Specify inclusion and exclusion criteria.	Section 2.2
Methods	6	Specify information sources and date last searched.	Section 2.3
Methods	7	Present full search strategies for all sources.	Supplementary Table S2
Methods	8	Describe the study selection process.	Section 2.4
Methods	9	Describe the data collection (charting) process.	Section 2.5
Methods	10	List and define all outcomes and variables sought.	Section 2.5
Methods	11	Specify methods used to assess risk of bias.	Section 2.6
Methods	13	Describe synthesis methods (mapping and narrative synthesis).	Sections 2.6 and 3
Results	16	Describe results of search and selection process with flow	Section 3; Figure 1

		diagram.	
Results	17	Report reasons for full-text exclusions.	Figure 1; Section 2.4
Results	18	Present characteristics of included studies.	Table 1; Section 3
Results	19	Present risk-of-bias assessments.	Table 2; Section 3
Discussion	23	Provide interpretation of findings in context.	Section 4
Discussion	24	Discuss limitations of evidence.	Sections 4.4–4.6
Discussion	26	Discuss implications for practice and future research.	Sections 4.4–4.6
Other Information	27	Provide registration, funding, and conflict-of-interest information.	Section 2.1; Funding/COI

Table S2. Full Reproducible Search Strategies.

Information Source	Platform	Date Last Searched	Time Span Covered	Exact Search Strategy	Filters Applied
PubMed / MEDLINE	PubMed	2026-02-28	2018-01-01 to 2026-01-15	("Burnout, Professional"[Mesh] OR burnout[tiab] OR "occupational burnout"[tiab]) AND ("Emergency Medical Services"[Mesh] OR "Intensive Care Units"[Mesh] OR emergency[tiab] OR "emergency department"[tiab] OR "acute care"[tiab] OR ICU[tiab] OR "critical care"[tiab] OR ED[tiab]) AND ("Health Personnel"[Mesh] OR "healthcare worker"[tiab] OR "healthcare workers"[tiab] OR "health care	English; Publication date limits applied

				worker"[tiab] OR "health care workers"[tiab] OR physician[tiab] OR physicians[tiab] OR nurse[tiab] OR nurses[tiab] OR paramedic[tiab] OR paramedics[tiab] OR clinician[tiab] OR clinicians[tiab]) AND ("Biomarkers"[Mesh] OR biomarker[tiab] OR biomarkers[tiab] OR cortisol[tiab] OR "heart rate variability"[tiab] OR HRV[tiab] OR "C-reactive protein"[tiab] OR CRP[tiab] OR S100B[tiab] OR cytokine[tiab] OR cytokines[tiab] OR interleukin[tiab] OR interleukins[tiab] OR "oxidative stress"[tiab] OR "allostatic load"[tiab]) AND ("2018/01/01"[dp] : "2026/01/15"[dp]) AND english[la]	
MDPI Platform Search	MDPI.com	2026-02-28	2018-01-01 to 2026-01-15	burnout AND biomarker AND emergency	English; Journal search filters
Backward Citation Searching	Manual screening	2026-02-28	Within included studies	Reference lists of included articles screened manually.	None
